# Cardioid oscillator-based pattern generator for imitating the time-ratio-asymmetrical behavior of the lower limb exoskeleton

**DOI:** 10.3389/fnbot.2024.1379906

**Published:** 2024-03-27

**Authors:** Qiang Fu, Tianhong Luo, TingQiong Cui, Xiangyu Ma, Shuang Liang, Yi Huang, Shengxue Wang

**Affiliations:** School of Intelligent Manufacturing Engineering, Chongqing University of Arts and Sciences, Chongqing, China

**Keywords:** cardioid oscillators, asymmetric time ratio, pattern generator, lower limb exoskeleton, invariant set

## Abstract

**Introduction:**

Periodicity, self-excitation, and time ratio asymmetry are the fundamental characteristics of the human gait. In order to imitate these mentioned characteristics, a pattern generator with four degrees of freedom is proposed based on cardioid oscillators developed by the authors.

**Method:**

The proposed pattern generator is composed of four coupled cardioid oscillators, which are self-excited and have asymmetric time ratios. These oscillators are connected with other oscillators through coupled factors. The dynamic behaviors of the proposed oscillators, such as phase locking, time ratio, and self-excitation, are analyzed via simulations by employing the harmonic balance method. Moreover, for comparison, the simulated trajectories are compared with the natural joint trajectories measured in experiments.

**Results and discussion:**

Simulation and experimental results show that the behaviors of the proposed pattern generator are similar to those of the natural lower limb. It means the simulated trajectories from the generator are self-excited without any additional inputs and have asymmetric time ratios. Their phases are locked with others. Moreover, the proposed pattern generator can be applied as the reference model for the lower limb exoskeleton controlling algorithm to produce self-adjusted reference trajectories.

## 1 Introduction

Lower limb exoskeletons (LLEs) are significant assist devices that can be used to improve the movement ability of people with walking disabilities by controlling the movement of exoskeleton joints in reference to the trajectories of the healthy human lower limbs (Wu et al., 2018; Pamungkas et al., 2019; Xue et al., 2019; Glowinski et al., 2020; Wei et al., 2020; Yihun et al., 2020; Ma et al., 2021). As the fitted trajectories from the gait data of the healthy human are invariant and non-adjustable, LLEs that are developed using trajectory tracking control methods cannot adjust their reference swing angle curves according to the environment changes (Nandi et al., 2008, 2009; Guo et al., 2010; Ekkachai and Nilkhamhang, 2016; Xu et al., 2016; Fu et al., 2017). On the contrary, the reference trajectory generated by the central pattern generators (CPGs), a biological neural circuit that generates rhythmic behaviors in animals, is periodical and self-excited. It is important to note that these trajectories possess the locked phase relationships and can be adjusted according to environment changes, thereby attracting significant research attention (Conradt, 2003; Acebrón et al., 2005; de Pina Filho et al., 2005; Morimoto et al., 2008; Saito et al., 2009; Katayama, 2012; Mora et al., 2012; Dingguo et al., 2017; Ferrario et al., 2018; Fu et al., 2018; Payam et al., 2018; Xie et al., 2019; Mokhtari et al., 2020; Pasandi et al., 2022; Wei et al., 2022).

Recently, CPG models, such as neuron-based CPG model (Saito et al., 2009; Katayama, 2012; Dingguo et al., 2017; Ferrario et al., 2018; Payam et al., 2018; Xie et al., 2019; Mokhtari et al., 2020; Pasandi et al., 2022; Wei et al., 2022) and oscillator-based CPG model (Conradt, 2003; Acebrón et al., 2005; de Pina Filho et al., 2005; Morimoto et al., 2008; Mora et al., 2012; Fu et al., 2018), are used for imitating the swing angles of human lower limbs. The former utilizes an oscillator to imitate the functions of neural cells, while the latter utilizes an oscillator to imitate periodic motions/torques. Therefore, the CPG models based on non-linear oscillators are more widely used for describing human walking than those based on neurons because of their simpler structure. Conradt (2003) proposed a CPG model based on the Kuramoto oscillator for a serpentine robot and achieved walking control during various environments. Morimoto et al. (2008) designed a CPG model of a humanoid robot and was able to simulate biped walking. Mora et al. (2012) designed a rhythmic gait generator for a mechanical walking apparatus by establishing a CPG model based on the van der Pol (VDP) oscillator and imitated hip and knee motions during walking. Nandi et al. (2008, 2009) proposed a CPG method for modeling a biped robot. de Pina Filho et al. (2005) applied a CPG model based on the Rayleigh oscillator for prosthesis control. However, the abovementioned CPG models are unable to precisely imitate asymmetric time ratios of human hip motions in their sagittal plane because they generate trajectories with a symmetrical time ratio. The asymmetric time ratios of hip joint mean that the duty of the forward progress is different from the duty of the backward progress within one period.

In this article, to achieve the asymmetric time ratio of the trajectories of human hip joints and to simulate the coupling relationship between human hip motion and knee motion, a cardioid oscillator based on a cardioid oscillator-based CPG (COCPG) model is designed by the authors (Fu et al., 2018) to imitate the swing angles of human lower limbs. The dynamic characteristics, such as the symmetry, self-excitation, astringency, and anti-interference of the COCPG, are numerically analyzed. Furthermore, the influence of the COCPG model's parameters on the frequency, amplitude, and offset are also numerically simulated and analyzed. Additionally, the trajectories generated by the COCPG model are compared with those experimentally measured from a tester in experiments and generated by the CPG model based on the Rayleigh oscillator.

## 2 Cardioid oscillator-based CPG model of lower limbs

### 2.1 Principle of the central pattern generator for lower limb exoskeleton

**Figure 1:**
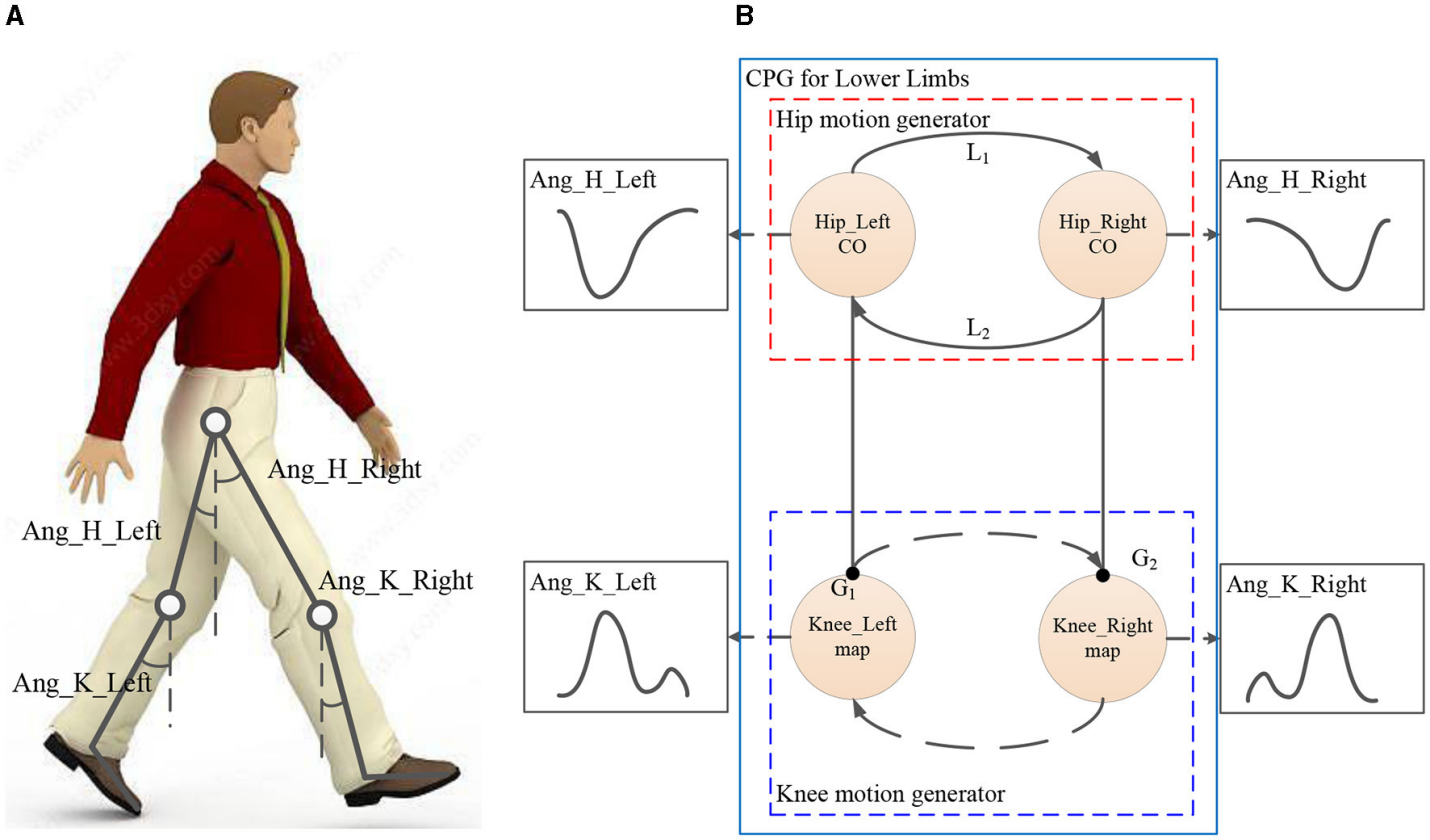
COCPG model for imitating the motions of lower limbs: **(A)** the simplified model of lower limbs and **(B)** the principle of the COCPG model.

For convenience, human lower limbs are usually considered as two double pendulums with four degrees of freedom (DOF) as shown in Figure 1A. As Figure 1A shows, the motions of the lower limbs include the swing angles of two hip joints and two knee joints, which are marked as *Ang*_*H*_*Right*, *Ang*_*H*_*Left*, *Ang*_*K*_*Right*, and *Ang*_*K*_*Left*, respectively. The motions of the lower limbs are asymmetric and coordinated, which means that four swing angles have asymmetric time ratios and pairwise coupling. Therefore, to imitate the motion of lower limbs, the COCPG model, whose principle is shown in Figure 1B, is proposed in this article.

As shown in Figure 1B, the proposed COCPG model is composed of a hip motion generator and a knee motion generator for imitating the motions of two hip joints and two knee joints, respectively. In particular, to imitate the behaviors of the Hip_Left joint and the Hip_Right joint, which are coupled with another, a hip motion generator is designed using two coupled cardioid oscillators (COs) through the oscillating terms *L*_1_ and *L*_2_ to generate the trajectories of *Ang*_*H*_*Right* and *Ang*_*H*_*Left* with locked phases. The cardioid oscillator shown in Figure 1B is an oscillator with an asymmetric limit cycle about the center proposed by the authors in a previous study (Fu et al., 2018). Correspondingly, the knee motion generator established by two mapping functions *G*_1_ and *G*_2_ generates two swing angles of the knee joints, *Ang*_*K*_*Right* and *Ang*_*K*_*Left*. Since the two mapping functions *G*_1_ and *G*_2_ are introduced from the Hip_Left CO and the Hip_Right CO, which are coupled, the functions *G*_1_ and *G*_2_ are coupled with one another.

### 2.2 Cardioid oscillator

The CO shown in Figure 1B is a non-linear oscillator whose limit cycle is a cardioid curve. Since the cardioid curve is a kind of asymmetric curve about its center, the CO can generate the trajectories with asymmetric time ratios.

#### 2.2.1 Designing of the cardioid oscillator

A dynamic system *A* can be written as


(1) $$\begin{array}{l} \{\begin{array}{c} {ẋ}_{1}=g_{1}\left(x_{1},x_{2}\right)\\ {ẋ}_{2}=g_{2}\left(x_{1},x_{2}\right) \end{array} \end{array}$$

where *x*_1_ and *x*_2_ are the states of the system *A*, *g*_1_(*x*_1_, *x*_2_) *and* *g*_2_(*x*_1_, *x*_2_) are the state equations on its states *x*_1_ and *x*_2_.

Assume that the set Ω is a non-zero set of the system *A*, which is given by


(2) $$\begin{array}{l} \Omega =\left\{\left(x_{1},x_{2}\right)|F\left(x_{1},x_{2}\right)=0\right\} \end{array}$$

where


(3) $$\begin{array}{l} C:F\left(x_{1},x_{2}\right)=0 \end{array}$$

is a closed cure of the system *A*.

According to the definition of the invariant set, we have


(4) $$\begin{array}{l} \left\{Ḟ\left(x_{1},x_{2}\right)=0|\left(x_{1},x_{2}\right)\in \Omega \right\}. \end{array}$$

Rewriting Equation (4) as


(5) $$\begin{array}{l} Ḟ\left(x_{1},x_{2}\right)=\frac{\partial F}{\partial x_{1}}{ẋ}_{1}+\frac{\partial F}{\partial x_{2}}{ẋ}_{2}=0. \end{array}$$

Substituting Equation (1) into Equation (5) leads to


(6) $$\begin{array}{l} \frac{\partial F}{\partial x_{1}}g_{1}\left(x_{1},x_{2}\right)+\frac{\partial F}{\partial x_{2}}g_{2}\left(x_{1},x_{2}\right)=0. \end{array}$$

Rewriting Equation (2) as


(7) $$\begin{array}{l} F\left(x_{1},x_{2}\right)={\left(x_{1}+a\right)}^{2}+{x_{2}}^{2}+bx_{2}+c{\left({x_{1}}^{2}+{x_{2}}^{2}\right)}^{2}\;\left(7\right) \end{array}$$

where *a*, *b*, *c* are the parameters for adjusting the asymmetric of the curve.

**Figure 2:**
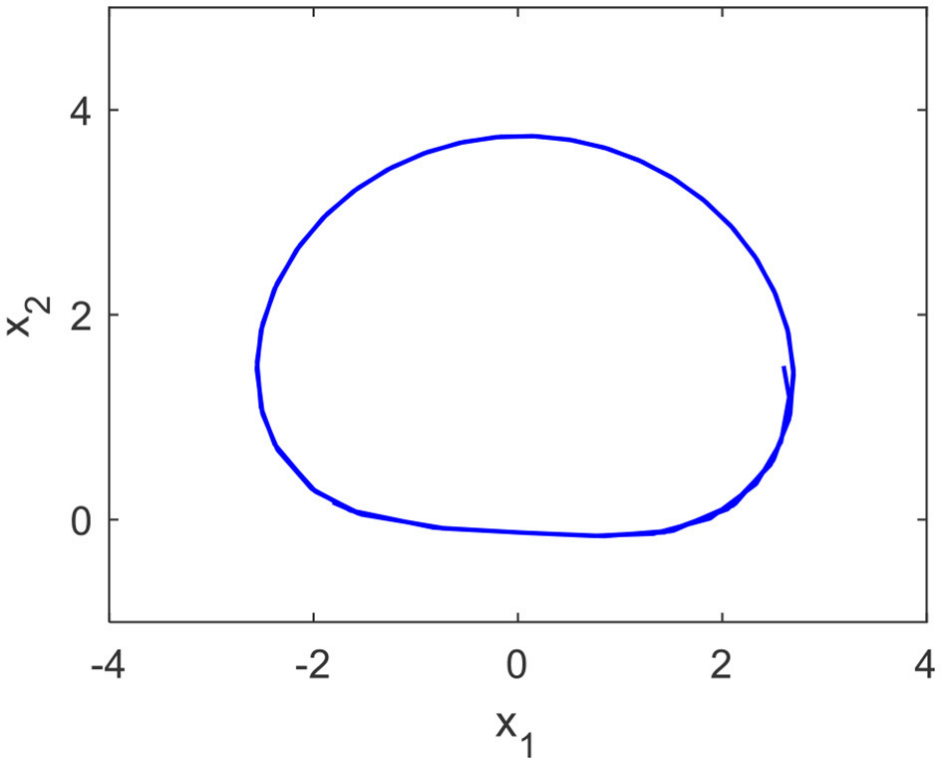
Cardioid curve with *a* = 5, *b* = 200, and *c* = −4.

When *a*, *b*, and *c* are equal to 5, 200, and −4, respectively, curve C in phase plane is shown in Figure 2.

Substituting Equation (7) into Equation (3) leads to


(8) $$\begin{array}{l} \Omega =\{\left(x_{1},x_{2}\right)|F\left(x_{1},x_{2}\right){\left(x_{1}+a\right)}^{2}+x_{2}{\;}^{2}\\ \;+bx_{2}+c{\left(x_{1}{\;}^{2}+x_{2}{\;}^{2}\right)}^{2}=0\}. \end{array}$$

According to Equation (8), defining *g*_1_(*x*_1_, *x*_2_) and *g*_2_(*x*_1_, *x*_2_) in Equation (1) as


(9) $$\begin{array}{l} \{\begin{array}{c} g_{1}\left(x_{1},x_{2}\right)=g_{11}+\gamma g_{12}F\left(x_{1},x_{2}\right)\\ g_{2}\left(x_{1},x_{2}\right)=g_{21}+\gamma g_{22}F\left(x_{1},x_{2}\right) \end{array} \end{array}$$

where *g*_11_, *g*_12_, *g*_21_, and *g*_22_ are the state equation of *x*_1_ and *x*_2_, γ is a positive constant for the convergence rate.

Substituting Equation (9) into Equation (6) gives


(10) $$\begin{array}{l} Ḟ\left(x_{1},x_{2}\right)=\left(\frac{\partial F}{\partial x_{1}}g_{11}+\frac{\partial F}{\partial x_{2}}g_{21}\right)+\left(\frac{\partial F}{\partial x_{1}}g_{12}+\frac{\partial F}{\partial x_{2}}g_{22}\right)\\ F\left(x_{1},x_{2}\right)=0. \end{array}$$

Since *F*(*x*_1_, *x*_2_) is equal to zero when (*x*_1_, *x*_2_) ∈ Ω, then Equation (10) can be rewritten as


(11) $$\begin{array}{l} \frac{\partial F}{\partial x_{1}}g_{11}+\frac{\partial F}{\partial x_{2}}g_{21}=0. \end{array}$$

The Lyapunov function *V* of the system *A* is given by


(12) $$\begin{array}{l} V=\frac{1}{2}{\left[{\left(x_{1}+a\right)}^{2}+{x_{2}}^{2}+bx_{2}+c{\left({x_{1}}^{2}+{x_{2}}^{2}\right)}^{2}\right]}^{2}. \end{array}$$

Differentiating Equation (12) and substituting Equations (1, 9) lead to


(13) $$\begin{array}{l} \dot{V}=\left(\frac{\partial F}{\partial x_{1}}g_{11}+\frac{\partial F}{\partial x_{2}}g_{21}\right)F\left(x_{1},x_{2}\right)+\left(\frac{\partial F}{\partial x_{1}}g_{12}+\frac{\partial F}{\partial x_{2}}g_{22}\right)\\ F^{2}\left(x_{1},x_{2}\right). \end{array}$$

According to Equations (11, 13), we have


(14) $$\begin{array}{l} \dot{V}=\gamma \left(\frac{\partial F}{\partial x_{1}}g_{12}+\frac{\partial F}{\partial x_{2}}g_{22}\right)F^{2}\left(x_{1},x_{2}\right). \end{array}$$

From Equation (12), it can be seen that when state (*x*_1_, *x*_2_) converges to infinity, *V* correspondingly converges to infinity.

When


(15) $$\begin{array}{l} \dot{V}\leq 0 \end{array}$$

$\left(x_{1},x_{2}\right)\to \left\{\left(x_{1},x_{2}\right)|\dot{V}=0\right\}$ as *t* → ∞.

Substituting Equation (14) into Equation (15) leads to


(16) $$\begin{array}{l} \frac{\partial F}{\partial x_{1}}g_{12}+\frac{\partial F}{\partial x_{2}}g_{22}\leq 0. \end{array}$$

According to Equations (11, 16), *g*_11_, *g*_12_, *g*_21_, and *g*_22_ can be given by


(17) $$\begin{array}{l} \{\begin{array}{c} g_{11}=-\frac{\partial F}{\partial x_{2}}\\ g_{12}=-\frac{\partial F}{\partial x_{1}}\\ g_{21}=\frac{\partial F}{\partial x_{1}}\\ g_{22}=-\frac{\partial F}{\partial x_{2}} \end{array}. \end{array}$$

Substituting Equation (17) into Equation (9), the system *A* can be written as


(18) $$\begin{array}{l} \{\begin{array}{c} {ẋ}_{1}=-\left[2x_{2}+b+2cx_{2}\left({x_{1}}^{2}+{x_{2}}^{2}\right)\right]\\ -\gamma \left[2x_{1}+2a+4cx_{1}\left({x_{1}}^{2}+{x_{2}}^{2}\right)\right]F\left(x_{1},x_{2}\right)\\ {ẋ}_{2}=\left[2x_{1}+2a+4cx_{1}\left({x_{1}}^{2}+{x_{2}}^{2}\right)\right]\\ -\gamma \left[2x_{2}+b+2cx_{2}\left({x_{1}}^{2}+{x_{2}}^{2}\right)\right]F\left(x_{1},x_{2}\right) \end{array}. \end{array}$$

Consequently, the curve C is the invariant set of the system *A* expressed by Equation (18).

#### 2.2.2 Coupling of cardioid oscillators

Two cardioid oscillators can be coupled via the coupling terms, which are represented by the following equations:


(19) $$\begin{array}{l} \{\begin{array}{c} {{ẋ}_{1}}^{i}=g_{1}\left({x_{1}}^{i},\;{x_{2}}^{i}\right)+\sigma^{i}\sum_{j=1}^{N}{L_{1}}^{ij}\left({x_{1}}^{i},\;{x_{2}}^{i},{x_{1}}^{j},\;{x_{2}}^{j}\right)\\ {{ẋ}_{2}}^{i}=g_{2}\left({x_{1}}^{i},\;{x_{2}}^{i}\right)-\sigma^{i}\sum_{j=1}^{N}{L_{2}}^{ij}\left({x_{1}}^{i},\;{x_{2}}^{i},{x_{1}}^{j},\;{x_{2}}^{j}\right) \end{array} \end{array}$$

where *i* and *j* are the sequences of the coupled oscillator; *N* is the count of the coupled COs; σ^*i*^ is the gain of the *i*th oscillator related to the coupling time; ${L_{1}}^{ij}\left({x_{1}}^{i},\;{x_{2}}^{i},{x_{1}}^{j},\;{x_{2}}^{j}\right)$ is the coupling gain between the state ${x_{1}}^{i}$ of the *i*th oscillator and the state ${x_{1}}^{j}$ of the *j*th oscillator, ${L_{2}}^{ij}\left({x_{1}}^{i},\;{x_{2}}^{i},{x_{1}}^{j},\;{x_{2}}^{j}\right)$ is the coupling gain between the state ${x_{2}}^{i}$ of the *i*th oscillator and the state ${x_{2}}^{j}$ of the *j*th oscillator, which are expressed by


(20) $$\begin{array}{l} \{\begin{array}{c} {L_{1}}^{ij}\left({x_{1}}^{i},\;{x_{2}}^{i},{x_{1}}^{j},\;{x_{2}}^{j}\right)=\left({x_{1}}^{j}+\;{x_{2}}^{j}\right){x_{2}}^{i}{x_{2}}^{i}\\ {L_{2}}^{ij}\left({x_{1}}^{i},\;{x_{2}}^{i},{x_{1}}^{j},\;{x_{2}}^{j}\right)=\left({x_{1}}^{j}+\;{x_{2}}^{j}\right){x_{1}}^{i}{x_{2}}^{i} \end{array}. \end{array}$$

### 2.3 CPG model for the lower limb exoskeleton

Coordinate transformation of Equation (18) yields


(21) $$\begin{array}{l} \left[\begin{array}{c} {x_{1}}^{\prime }\\ {x_{2}}^{\prime }\\ 1 \end{array}\right]=\left[\begin{array}{ccc} D_{11} & 0 & P_{1}\\ 0 & D_{22} & P_{2}\\ 0 & 0 & 1 \end{array}\right]\left[\begin{array}{c} x_{1}\\ x_{2}\\ 1 \end{array}\right] \end{array}$$

where $\left[\begin{array}{ccc} D_{11} & 0 & P_{1}\\ 0 & D_{22} & P_{2}\\ 0 & 0 & 1 \end{array}\right]$ is the homogeneous transfer matrix, *D*_11_ and *D*_22_ are the scale coefficients, and *P*_1_ and *P*_2_ are the translate coefficients. We have


(22) $$\begin{array}{l} \left[\begin{array}{c} {\dot{x_{1}}}^{\prime }\\ {\dot{x_{2}}}^{\prime } \end{array}\right]\left[\begin{array}{cc} D_{11} & 0\\ 0 & D_{22} \end{array}\right]\left[\begin{array}{c} \dot{x_{1}}\\ \dot{x_{2}} \end{array}\right]. \end{array}$$

Substituting Equations (21, 22) into Equations (18, 19, 20), and separating the frequency parameter ω yields


(23) $$\begin{array}{l} \{\begin{array}{c} {\dot{x_{1}}}^{\prime }=-\frac{k\left\{\left[2{x_{2}}^{\prime }+b-2c{x_{2}}^{\prime }\left({{x_{1}}^{\prime }}^{2}+{{x_{2}}^{\prime }}^{2}\right)\right]-k\gamma \left[2{x_{1}}^{\prime }+2a-4c{x_{1}}^{\prime }\left({{x_{1}}^{\prime }}^{2}+{{x_{2}}^{\prime }}^{2}\right)\right]F\left({x_{1}}^{\prime },\;{x_{2}}^{\prime }\right)\right\}+\sigma^{i}\left({{x_{1}}^{\prime }}^{j}+\;{{x_{2}}^{\prime }}^{j}\right){{x_{2}}^{\prime }}^{i}{{x_{2}}^{\prime }}^{i}}{D_{11}\omega }\\ {\dot{x_{2}}}^{\prime }=\frac{k\left\{\left[2{x_{1}}^{\prime }+2a-4c{x_{1}}^{\prime }\left({{x_{1}}^{\prime }}^{2}+{{x_{2}}^{\prime }}^{2}\right)\right]-k\gamma \left[2{x_{2}}^{\prime }+b-2c{x_{2}}^{\prime }\left({{x_{1}}^{\prime }}^{2}+{{x_{2}}^{\prime }}^{2}\right)\right]F\left({x_{1}}^{\prime },\;{x_{2}}^{\prime }\right)\right\}-\sigma^{i}\left({{x_{1}}^{\prime }}^{j}+\;{{x_{2}}^{\prime }}^{j}\right){{x_{1}}^{\prime }}^{i}{{x_{2}}^{\prime }}^{i}}{D_{22}\omega } \end{array}. \end{array}$$

Considering the states ${x_{1}}^{\prime }$ and ${x_{2}}^{\prime }$ of the CO as the hip angle and velocity of the CPG model, respectively, we have


(24) $$\begin{array}{l} {{Ang\text{\_}H=x}_{1}}^{\prime } \end{array}$$

(25) $$\begin{array}{l} {{Vel\text{\_}H=x}_{2}}^{\prime }. \end{array}$$

Fitting the hip angle and the knee angle of a natural gait, the mapping function *G* between the hip motion and the knee motion can be defined as


(26) $$\begin{array}{l} G\left(\;Ang\text{\_}H\;,Vel\text{\_}H\right)={\left(Vel\text{\_}H-Ang\text{\_}H+\theta \right)}^{2} \end{array}$$

where θ is a constant.

From Equations (22–26), the COCPG model can be expressed as


(27) $$\begin{array}{l} \{\begin{array}{c} {\dot{x_{1}}}^{\prime }=-\frac{k\left\{\left[2{x_{2}}^{\prime }+b-2c{x_{2}}^{\prime }\left({{x_{1}}^{\prime }}^{2}+{{x_{2}}^{\prime }}^{2}\right)\right]-k\gamma \left[2{x_{1}}^{\prime }+2a-4c{x_{1}}^{\prime }\left({{x_{1}}^{\prime }}^{2}+{{x_{2}}^{\prime }}^{2}\right)\right]F\left({x_{1}}^{\prime },\;{x_{2}}^{\prime }\right)\right\}+\sigma \left({x_{3}}^{\prime }+\;{x_{4}}^{\prime }\right){{x_{2}}^{\prime }}^{2}}{D_{11}\omega }\\ {\dot{x_{2}}}^{\prime }=\frac{k\left\{\left[2{x_{1}}^{\prime }+2a-4c{x_{1}}^{\prime }\left({{x_{1}}^{\prime }}^{2}+{{x_{2}}^{\prime }}^{2}\right)\right]-k\gamma \left[2{x_{2}}^{\prime }+b-2c{x_{2}}^{\prime }\left({{x_{1}}^{\prime }}^{2}+{{x_{2}}^{\prime }}^{2}\right)\right]F\left({x_{1}}^{\prime },\;{x_{2}}^{\prime }\right)\right\}-\sigma \left({x_{3}}^{\prime }+\;{x_{4}}^{\prime }\right){x_{1}}^{\prime }{x_{2}}^{\prime }}{D_{22}\omega }\\ {\dot{x_{3}}}^{\prime }=-\frac{k\left\{\left[2{x_{4}}^{\prime }+b-2c{x_{4}}^{\prime }\left({{x_{3}}^{\prime }}^{2}+{{x_{4}}^{\prime }}^{2}\right)\right]-k\gamma \left[2{x_{3}}^{\prime }+2a-4c{x_{3}}^{\prime }\left({{x_{3}}^{\prime }}^{2}+{{x_{4}}^{\prime }}^{2}\right)\right]F\left({x_{3}}^{\prime },\;{x_{4}}^{\prime }\right)\right\}+\sigma \left({x_{1}}^{\prime }+\;{x_{2}}^{\prime }\right){{x_{4}}^{\prime }}^{2}}{D_{11}\omega }\\ {\dot{x_{4}}}^{\prime }=\frac{k\left\{\left[2{x_{3}}^{\prime }+2a-4c{x_{3}}^{\prime }\left({{x_{3}}^{\prime }}^{2}+{{x_{4}}^{\prime }}^{2}\right)\right]-k\gamma \left[2{x_{4}}^{\prime }+b-2c{x_{4}}^{\prime }\left({{x_{3}}^{\prime }}^{2}+{{x_{4}}^{\prime }}^{2}\right)\right]F\left({x_{3}}^{\prime },\;{x_{4}}^{\prime }\right)\right\}-\sigma \left({x_{1}}^{\prime }+\;{x_{2}}^{\prime }\right){x_{3}}^{\prime }{x_{4}}^{\prime }}{D_{22}\omega }\\ Ang\text{\_}H\text{\_}Right={x_{1}}^{\prime }\\ Ang\text{\_}K\text{\_}Right={\left(0.05\left({x_{2}}^{\prime }-0.8{x_{1}}^{\prime }\right)+\theta \right)}^{2}+P_{3}\\ Ang\text{\_}H\text{\_}Left={x_{3}}^{\prime }\\ Ang\text{\_}K\text{\_}Left={\left(0.05\left({x_{4}}^{\prime }-0.8{x_{3}}^{\prime }\right)+\theta \right)}^{2}+P_{3} \end{array}. \end{array}$$

As Equations (11, 22, 27) show, the parameters *a*, *b*, and *c* of the COCPG model can be used to adjust the shape of the limit cycle, γ can be used to adjust the convergence rate of the trajectory generated by the COCPG model, ω can be used to adjust the frequency, *D*_11_ and *D*_22_ are used to adjust the amplitude, and *P*_1_ and *P*_3_ can be used to adjust the offset. Therefore, by choosing appropriate values of the mentioned parameters, the rhythmic motions of lower limbs with different frequencies and amplitudes can be imitated by the proposed COCPG model given by Equations (21, 22, 27).

## 3 Numerical simulations and analyses

### 3.1 Behaviors of the cardioid oscillator

In this section, the behaviors of the CO, such as asymmetry, self-excited oscillation, anti-interference, and convergence, are analyzed using numerical simulations.

#### 3.1.1 Asymmetry

**Figure 3:**
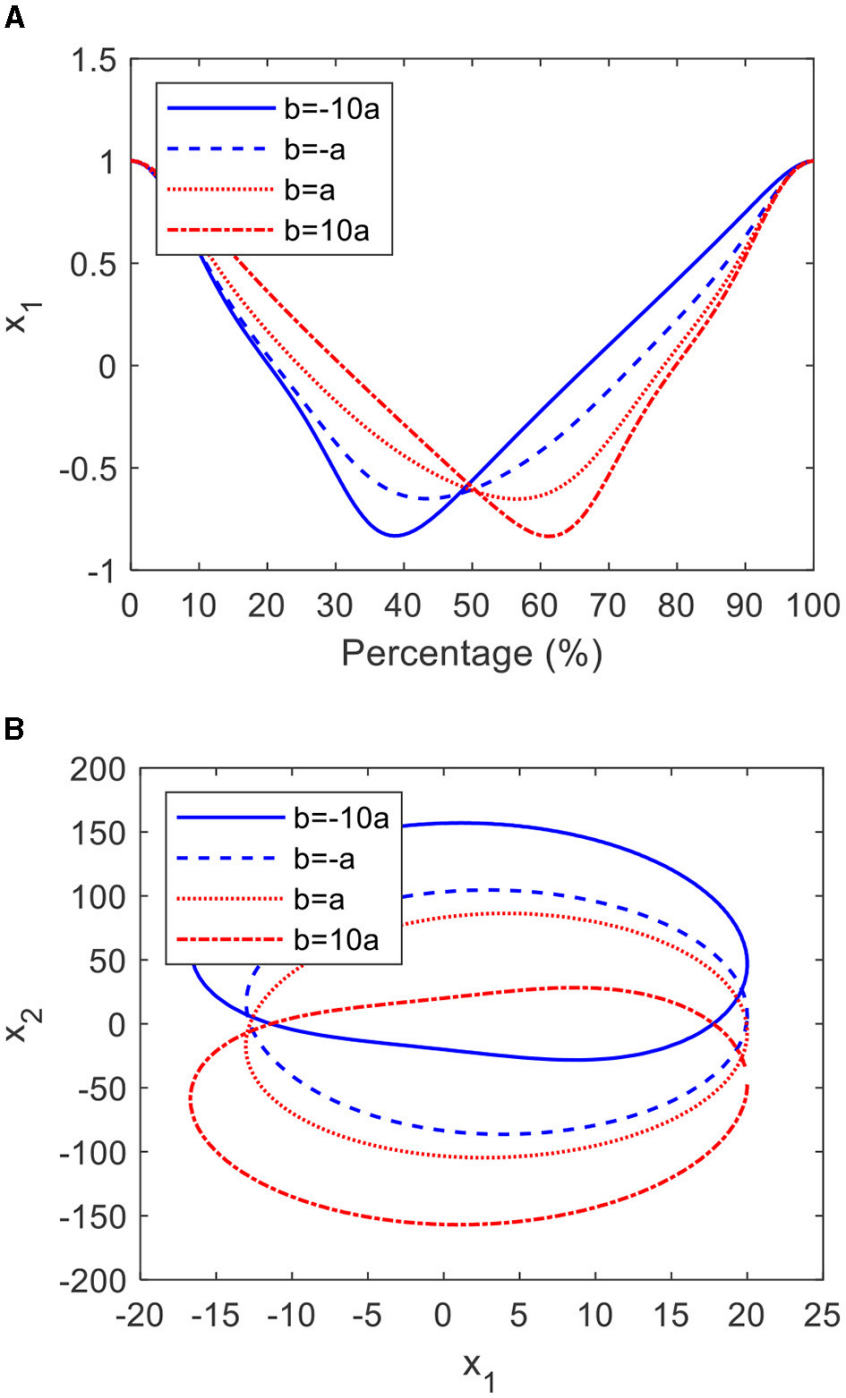
The cardioid oscillator with verified *b*: **(A)** the graphs and **(B)** the limit cycles.

From Equation (18), it is clear that the shape of the CO can be adjusted through parameters *a* and *b*. When *a* equal to 5, *c* equal to −4, and *b* equal to −50, −5, 5, 50, respectively, the graphs and phase planes are shown in Figure 3. It can be seen from Figure 3A that as *b* increases from −50 to 50, the proportion of the forward progress significantly increases by 24%. Meanwhile, the shape of the limit cycle becomes increasingly asymmetric, as shown in Figure 3B.

#### 3.1.2 Anti-interference and self-excitement

**Figure 4:**
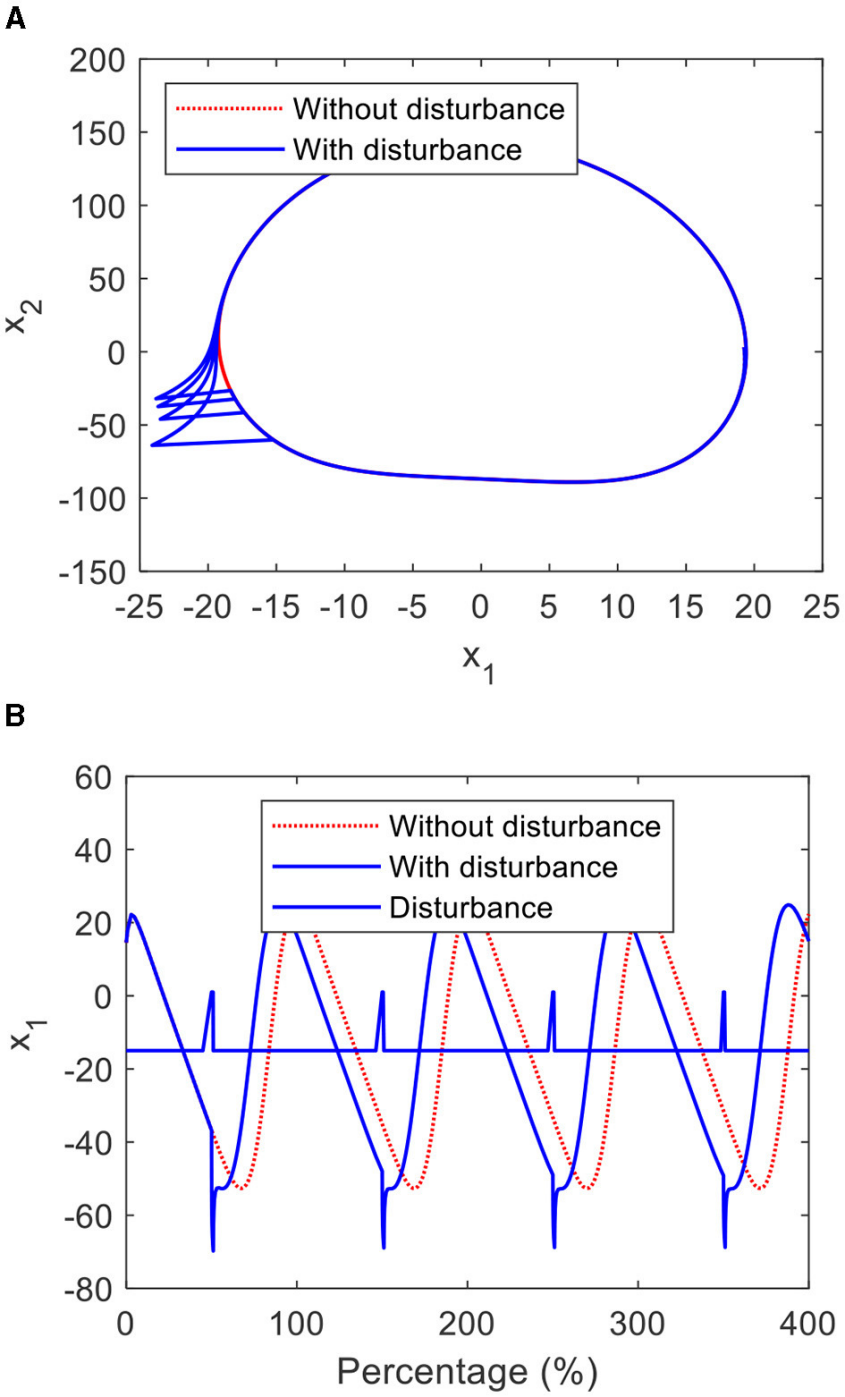
Limit cycles and graphs of the CO: **(A)** the limit cycles and **(B)** the graphs.

**Figure 5:**
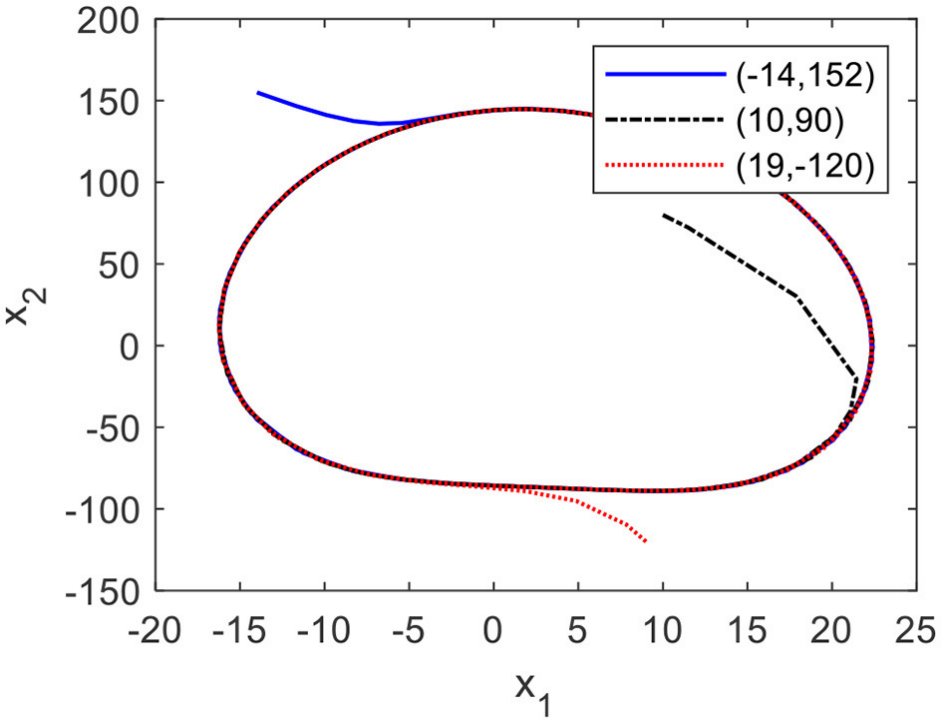
Limit cycles of the CO with different initial conditions.

Figures 4A, B show the phase plane and graph of the CO, which is perturbed by a disturbance signal with the amplitude of 45. From Figure 4, it is clear that the states of the CO deviate from the limit cycle when the CO is disturbed by the pulse signal and return to the limit cycle in a short time. From Figure 5, it can be seen that when the initial conditions of the CO equal to (−14, 152), (10, 90), and (19,−120), their states autonomously converge to the limit cycle, even though these initial conditions deviate from the limit cycle.

The results indicate that the CO has a stable limit circle to lead a stable trajectory of the lower limb.

#### 3.1.3 Convergence rate of the cardioid oscillator

**Figure 6:**
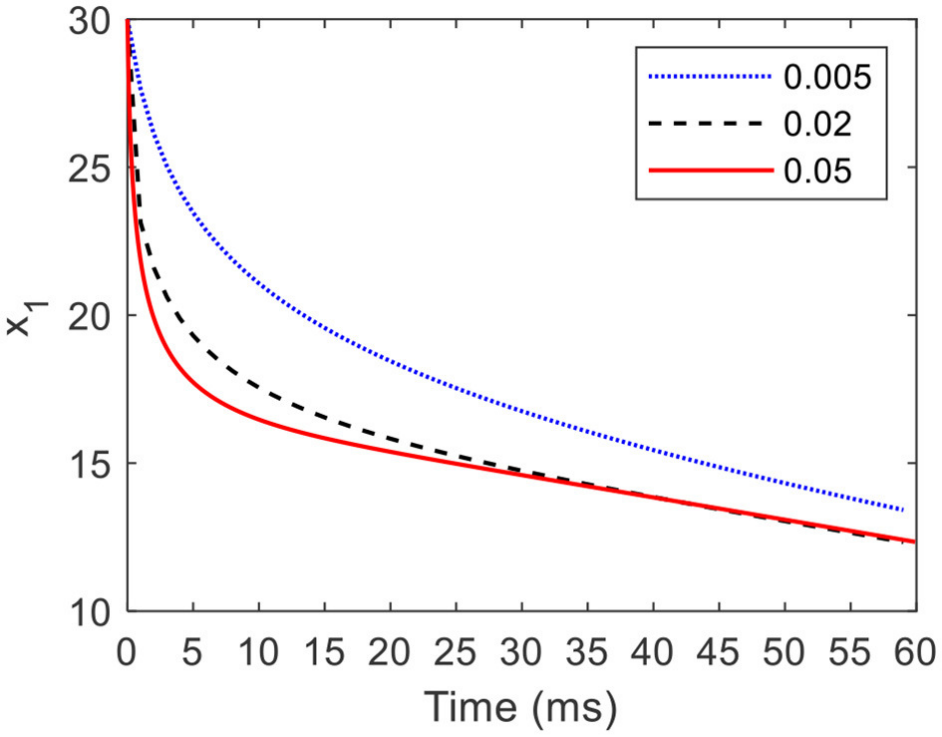
Partial graphs of the CO with different γ* values*.

According to Equation (18), when an initial condition is (30, 0), *a* = 5, *b* = 200, and *c* = −4, Figure 6 shows the partial graphs of the CO with γ of 0.005, 0.02, and 0.05. When γ is equal to 0.005, it takes 15 ms for state *x*_1_ converging from the initial condition where *x*_1_=30 to the limit cycle where *x*_1_=20. As γ increases to 0.02, the required time of the converging process decreases to 5 ms. Furthermore, when γ is increases to 0.05, the required time of the converging process decreases to 2 ms.

The results indicate that the COCPG model provides a strong response to the environment changes.

### 3.2 Numerical simulations and analyses of the COCPG model

In this section, when modeling the motions of lower limbs with different frequencies and amplitudes, the influence of the parameters of the COCPG model on the frequency, amplitude, and offset of the trajectories generated by the COCPG model are numerically simulated and analyzed. When modeling the motions of lower limbs, the parameters *a*, *b*, and *c* in Equation (27) are equal to 5, 200, and 2, respectively, *k* is 0.64, and γ is 0.02.

#### 3.2.1 Frequency

**Figure 7:**
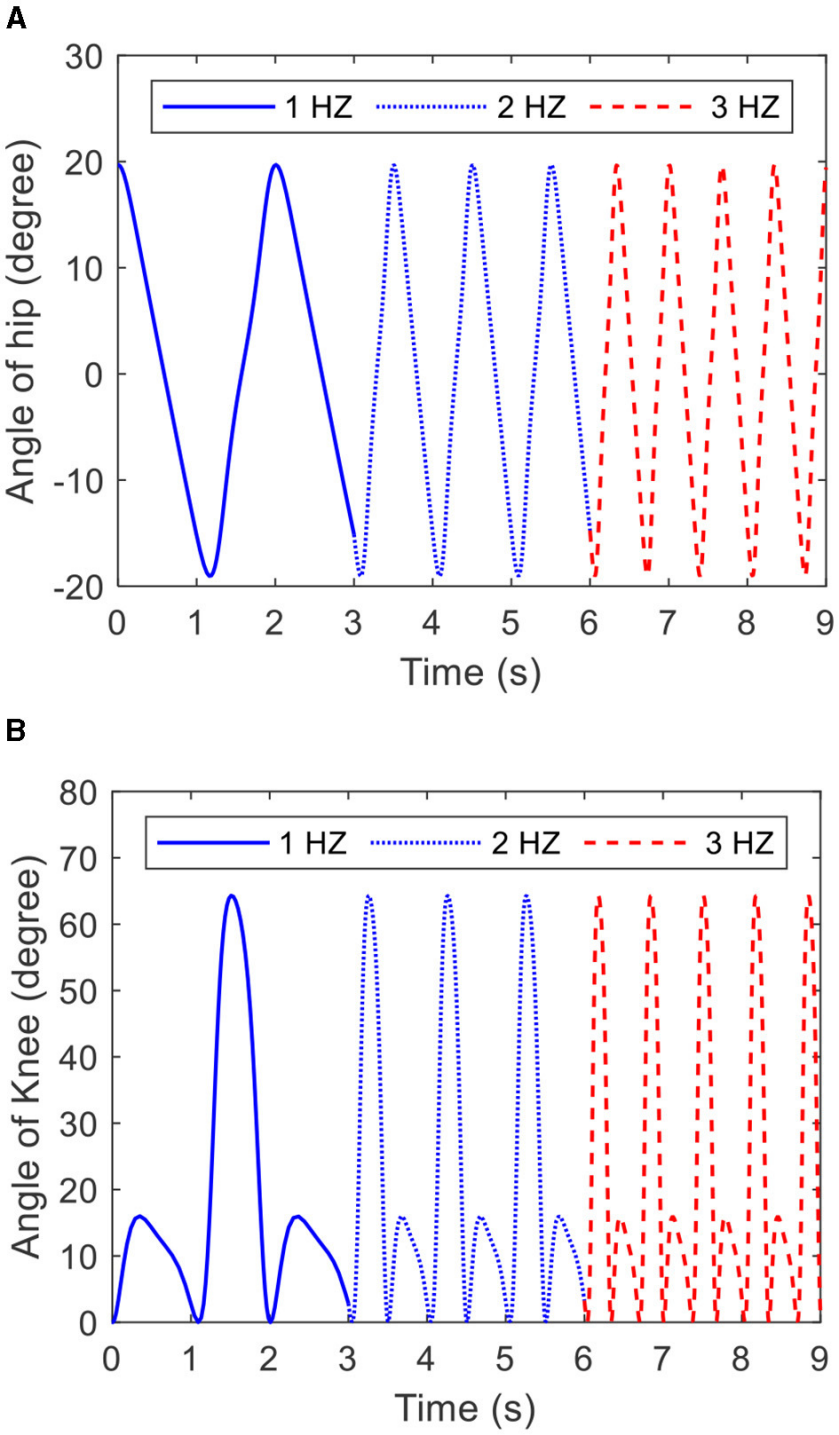
Trajectories of the joints of the lower limb generated by the COCPG model with different ω: **(A)** the hip joint and **(B)** the knee joint.

As Equation (27) shows, the frequency of the COCPG model is determined by ω. Figure 7 depicts the trajectories of the hip and knee joints generated by the COCPG model with ω of 1, 2, and 3 Hz, respectively. From Figure 7, it is obvious that the frequency of the COCPG model increases with increasing ω. Therefore, the frequency of the COCPG model is determined by the value of ω.

#### 3.2.2 Amplitude

**Figure 8:**
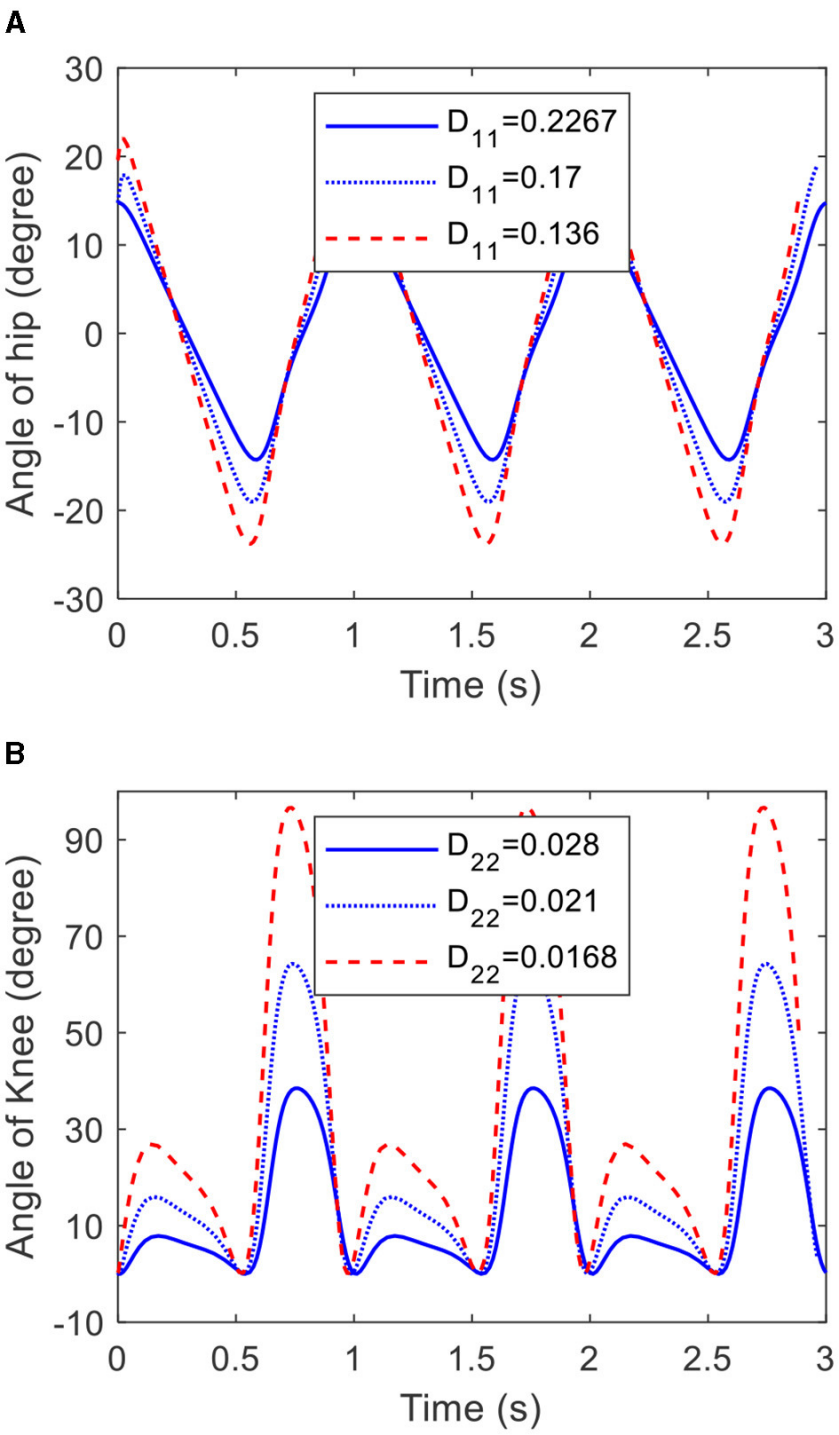
Trajectories of the hip and knee joints generated by the COCPG model with different *D*_11_ and *D*_22_: **(A)** the hip joint and **(B)** the knee joint.

From Equation (27), it can be inferred that the angle amplitude of the hip joint of the COCPG model is only determined by *D*_11_, and the angle amplitude of the knee joint of the COCPG model is determined by *D*_11_ and *D*_22_. Figure 8 shows the trajectories of the joints of the lower limb generated by the COCPG model with different *D*_11_ and *D*_22_. Figure 8A shows the trajectories of the hip joint generated by the COCPG model when *D*_11_ is equal to 0.2267, 0.17, and 0.136, respectively. Figure 8B shows the trajectories of the knee joint generated by the model with *D*_11_ of 0.17 when *D*_22_ is equal to 0.028, 0.021, and 0.0168, respectively. From Figure 8, it is obvious that the amplitudes of the trajectories generated by the COCPG model decrease with increasing *D*_11_ and *D*_22_. Therefore, the trajectories of the joints of the lower limb with different amplitudes can be achieved by adjusting *D*_11_ and *D*_22_.

#### 3.2.3 Offset

**Figure 9:**
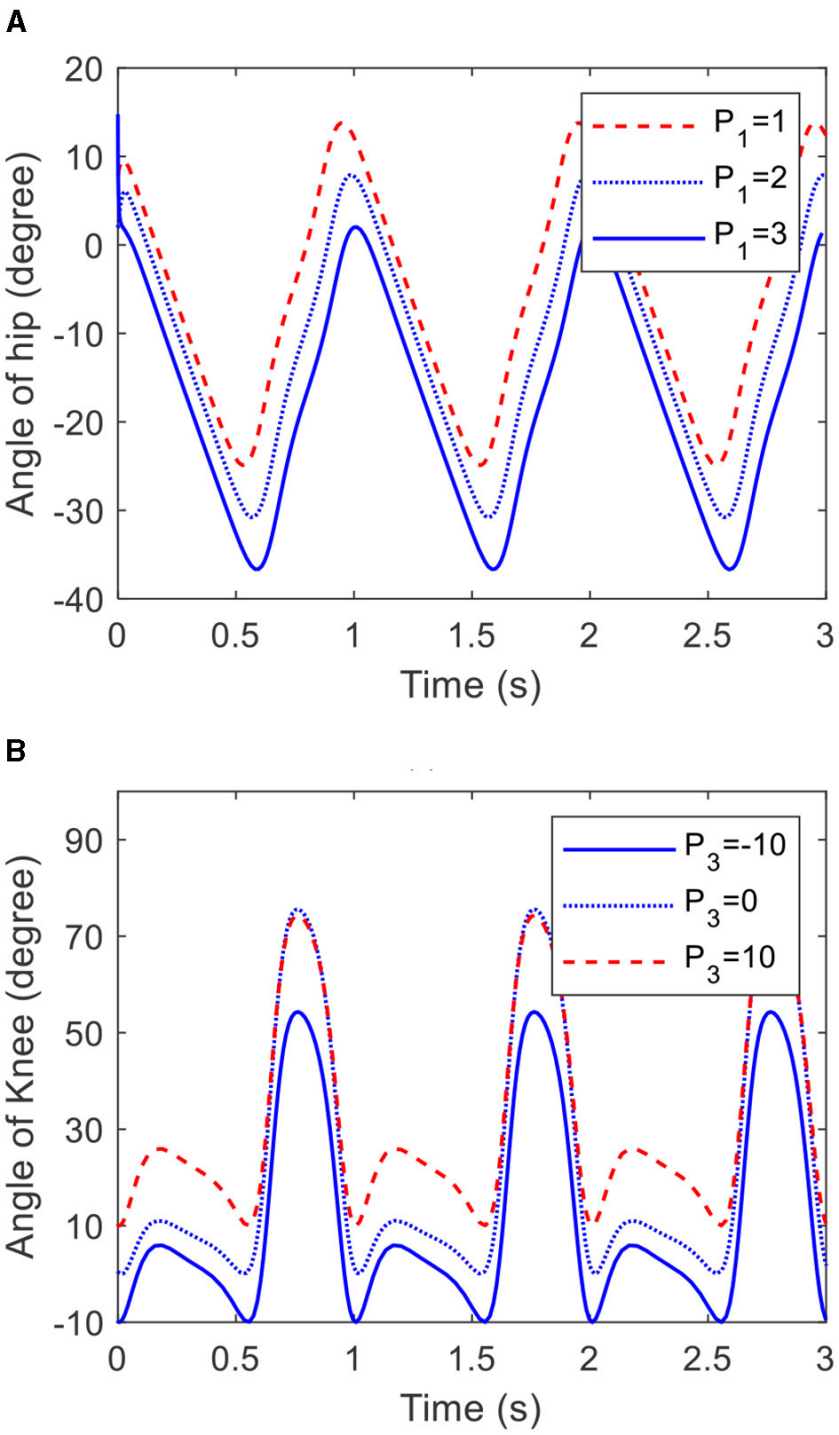
Trajectories of the hip and knee joints generated by the COCPG model with different *P*_1_ and *P*_3_: **(A)** the hip joint and **(B)** the knee joint.

Going by Equation (27), the offset of the trajectories generated by the COCPG model are determined by *P*_1_ and *P*_3_. Figure 9 shows the trajectories of the joints of the lower limb generated by the COCPG model with different *P*_1_ and *P*_3_, respectively. Figure 9A shows the trajectories of the hip joint generated by the COCPG model with *P*_1_ of 1, 2, and 3, and Figure 9B shows the trajectories of the knee joint generated by the COCPG model with *P*_3_ of −10, 0, and 10. It is clearly seen from Figure 9 that the offsets of the trajectories of the joints of the lower limb generated by the COCPG model increases along with the increase in the absolute value of *P*_1_ and *P*_3_.

## 4 Experiments and results

### 4.1 Experimental setup

**Figure 10:**
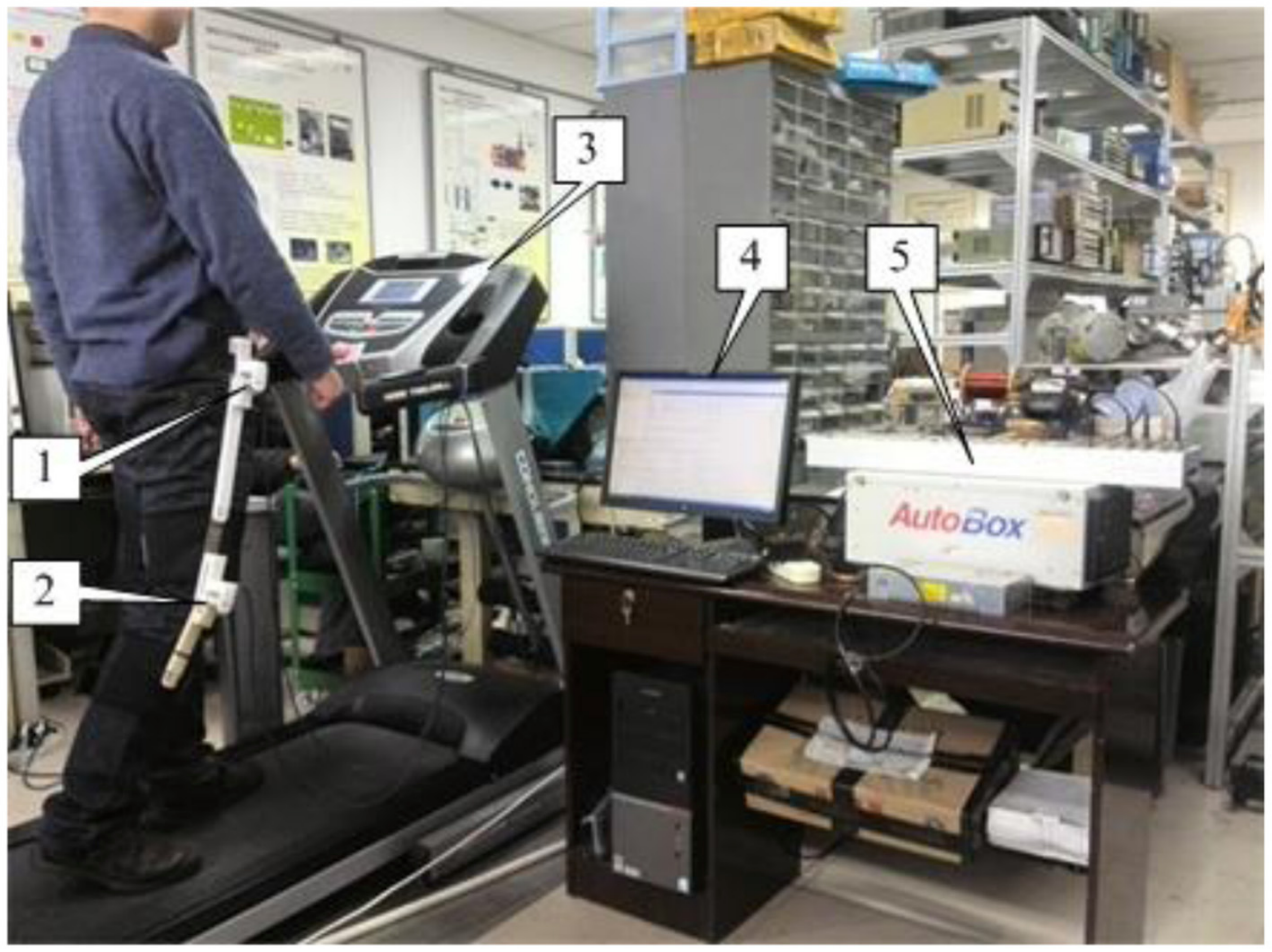
Test platform updated from Wang et al. (2013) [1-angle sensor (AS3); 2-angle sensor (AS4); 3-treadmill; 4-PC; 5-dSPACE DS1103].

In order to evaluate the modeling accuracy of the COCPG model proposed in this article, the trajectories of the joints of the lower limb during walking are measured on an experimental setup, which is a test platform for prosthetic knees built by the authors (Wang et al., 2013). The test platform is presented in Figure 10. As shown in Figure 10, the experimental setup comprises a treadmill, two angle sensors, a data acquisition unit, and a tester. Two angle potentiometers, which are attached on the hip joint and the knee joint of the tester by three linkages, are used for measuring the angular displacements of the hip and knee joints. The host computer interrogates the voltage signals from the potentiometers via the data acquisition unit (type: DS1103, from the dSPACE GmbH, Germany) with a sampling frequency of 1 kHz.

### 4.2 Results and analysis

**Figure 11:**
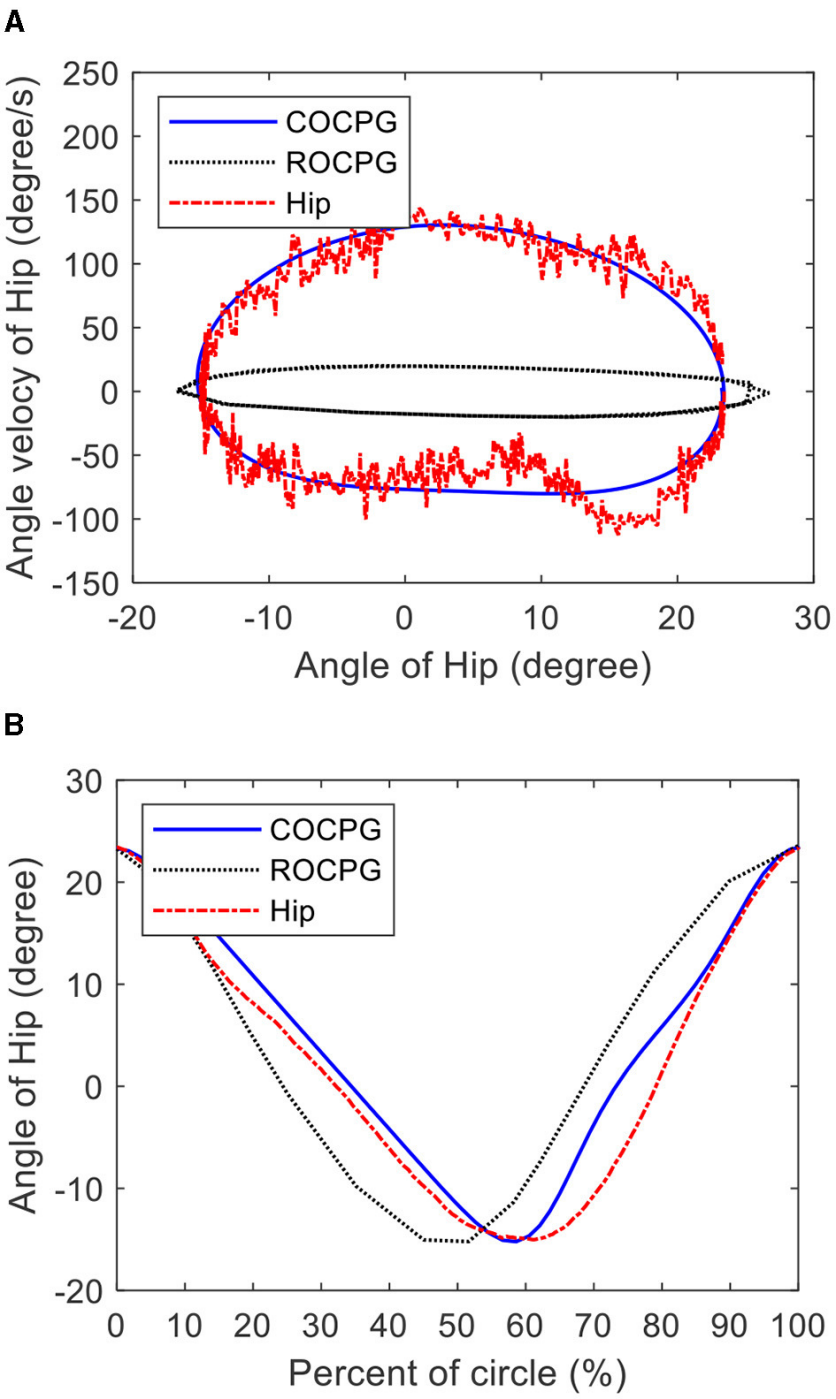
Hip motions measured from the tester on the test platform and generated by the COCPG model and the ROCPG model: **(A)** the limit cycles and **(B)** the trajectories.

Figures 11A, B show the limit cycles and trajectories of the hip joint generated by the COCPG model (Equation 27) and measured from the tester on the test platform. For comparison, the limit cycle and trajectory of the hip joint generated by the CPG model based on the Rayleigh oscillator (ROCPG) are simultaneously provided. The Rayleigh oscillator is expressed as (de Pina Filho et al., 2005)


(28) $$\begin{array}{l} ÿ+d\left(l-{ẏ}^{2}\right)ẋ+k^{2}y=0 \end{array}$$

where *y* is the output of the Rayleigh oscillator, *d*, *l*, and *k* are the parameters of the Rayleigh oscillator. In this article, the ROCPG model is based on Equation (28) with *d* = 0.14, *l* = 0.013, and *k* = 1.

Figure 11A shows that the limit cycle of the COCPG model is an asymmetric closed curve about its center, which is consistent with the measured limit cycle of the hip joint of the tester. However, the limit cycle of the ROCPG model is a symmetrical closed curve about its center, which differs from the measured limit cycle of the hip joint of the tester. Additionally, as shown in Figure 11B, the forward trajectory of the hip joint of the COCPG model accounts for 60% of one period and the forward progress of the hip joint generated by the ROCPG model accounts for 50%. Thus, the trajectory of the hip joint of the COCPG model is closer to that of the natural gait than that of the ROCPG model. Therefore, compared with the ROCPG model, the COCPG model is able to accurately imitate the rhythmic motions of the hip joints.

**Figure 12:**
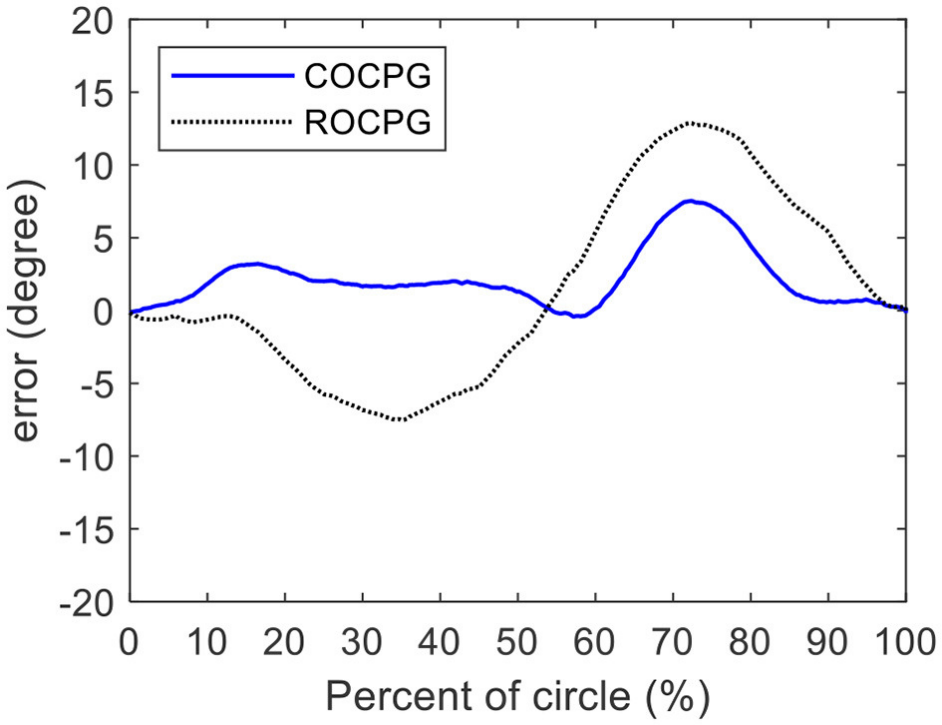
Differences between the measured trajectory of the hip joint of the tester and the trajectories of the hip joint of the COCPG model and the ROCPG model.

Figure 12 shows the differences between the measured trajectory of the hip joint of the tester and the trajectories of the hip joints generated by the COCPG and the ROCPG models. These differences can be defined as modeling errors. As shown in Figure 12, the maximum modeling error of the motion trajectory of the hip joint of the COCPG model is about 5°. Meanwhile, the maximum modeling error of the ROCPG model is about 15°, which is three times that of the COCPG model. The main source of the modeling error of the COCPG model is the flexible binding method of the measure unit. On the contrary, the main source of the modeling error of the ROCPG model is the asymmetry of the limit circle of the Rayleigh oscillator. As a result, the modeling accuracy of the output trajectories of the hip joint of the COCPG model is higher than that of the ROCPG model.

**Figure 13:**
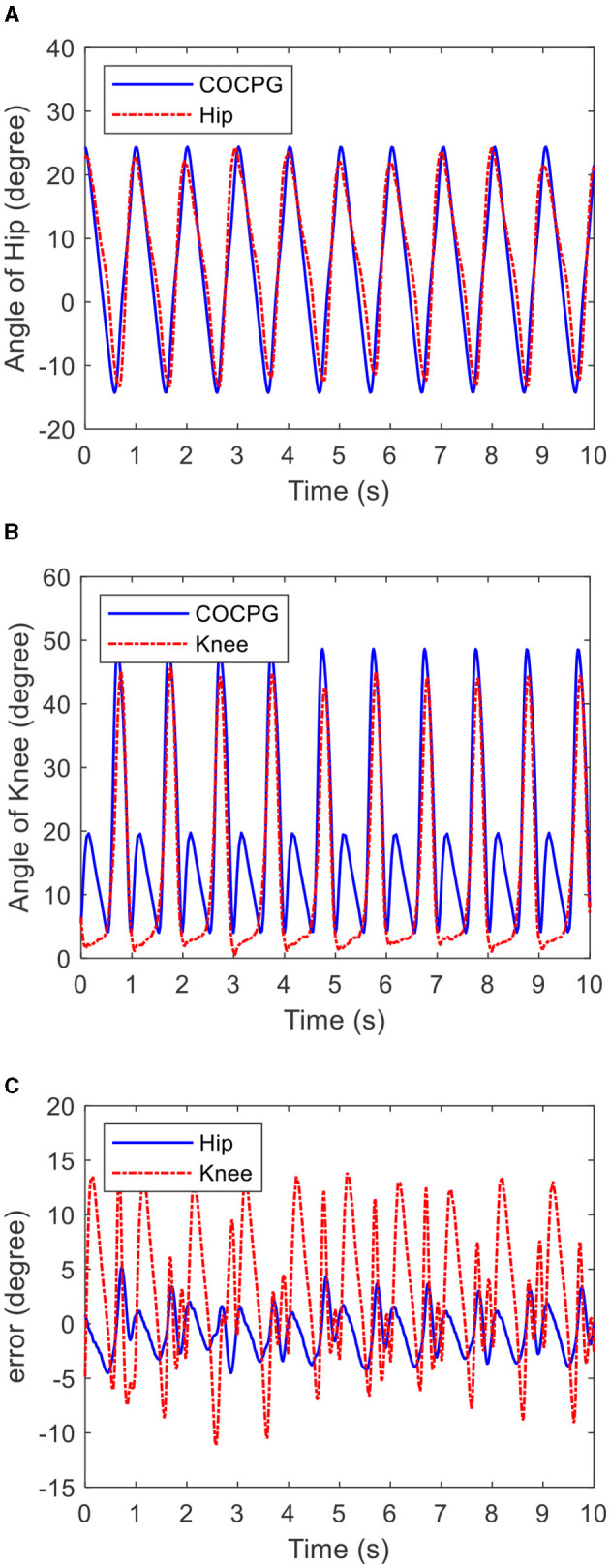
Time histories of the multi-period trajectories of the lower limb generated by the COCPG model and measured from the tester on the test platform: **(A)** the trajectories of the hip joint, **(B)** the trajectories of the knee joint, and **(C)** differences between the measured trajectories of the tester and the COCPG model.

Figure 13 shows the time histories of the multi-periodic trajectories of the lower limb generated by the COCPG model and measured from the tester on the test platform. Figure 13A shows the trajectories of the hip joint and Figure 13B shows the trajectories of the knee joint. Figure 13C shows the differences between the measured trajectories of the tester and generated trajectories of the COCPG model. The maximum error between the hip joint trajectories and the models is 5°, while the maximum error between the knee joint trajectories and the models is 15°. The maximum error of the hip joint appears at the backward of the swing phase, and the maximum error of the knee joint appears at the stance phase. From Figure 13, it is clear that the trajectories of the COCPG model are consistent with those of the measured trajectories. Therefore, the COCPG model is suitable for imitating the rhythmic motions of the hip joint of the lower limb. However, the modeling error of the trajectory of the knee joint with the COCPG model still exists, which may be because the posture of the tester is deformed while walking on the treadmill and/or the angle potentiometers are not assembled on the sagittal plane.

## 5 Conclusions

In this article, to achieve the asymmetric time ratio of the trajectories of human hip joints and to simulate the coupling relationship between hip motions and knee motions, based on the CO with a central asymmetric limit cycle, the COCPG model for simulating asymmetric swing angles of lower limbs is proposed and developed. Based on the proposed method, the behaviors, such as frequency, amplitude, and offset, are analyzed by simulations. Additionally, in order to verify the accuracy of the COCPG model, experiments are conducted for comparing the outputs of the COCPG model with the measured trajectories from the tester on the test platform. The research results show that the trajectories of the hip joint generated by the COCPG model proposed in this article follow the asymmetric time ratio. The time ratios of the trajectories of the COCPG varied from 38 to 62%. Meanwhile, the time ration of the trajectories of the ROCPG are invariable. Compared with the ROCPG model, the COCPG model can imitate the hip motion with higher accuracy and the trajectories of the knee joint generated by the COCPG model is coupled with the hip motion. The maximum modeling error of the COCPG is 5°, which is introduced from the binding method of the measure unit. On the contrary, as a result of the symmetric limit circle, the maximum modeling error of the ROCPG increases to 15°. Moreover, the motion of the lower limb with different frequencies and amplitudes can be achieved by adjusting the parameters of the COCPG model. Therefore, the proposed pattern generator can be applied as the reference model for the lower limb exoskeleton controlling algorithm to produce the self-adjusted reference trajectories.

Although the proposed COCPG is an effective model for imitating the asymmetric and coupled behaviors of the lower limb, the coupling between the COCPG model and humans should be studied to promote its application in controlling a lower limb exoskeleton.

## Data availability statement

The original contributions presented in the study are included in the article/supplementary material, further inquiries can be directed to the corresponding author.

## Author contributions

QF: Writing – review & editing, Writing – original draft. TL: Writing – review & editing. TC: Writing – review & editing, Formal analysis, Data curation. XM: Writing – review & editing, Validation, Project administration. SL: Writing – review & editing, Resources, Formal analysis. YH: Writing – review & editing, Data curation, Conceptualization. SW: Writing – review & editing, Resources.
